# Medical students’ and health professionals’ knowledge regarding acute kidney injury: a cross-sectional study in the city of São Paulo, Brazil

**DOI:** 10.1080/0886022X.2022.2131575

**Published:** 2022-11-03

**Authors:** Farid Samaan, Danilo Aoike, Ricardo Pagrion-Neto, Thiago Cesar Pons, Rafaela Bracci Lisboa, Emmanuel A. Burdmann

**Affiliations:** a Instituto Dante Pazzanese de Cardiologia, São Paulo, Brazil; b LIM 12, Disciplina de Nefrologia, Universidade de São Paulo, São Paulo, Brazil; c Disciplina de Nefrologia, Universidade Federal de São Paulo, Brazil; d Universidade Nove de Julho, São Paulo, Brazil

**Keywords:** Acute kidney injury, knowledge, early diagnosis, disease prevention

## Abstract

**Background:**

Acute kidney injury (AKI) is a worldwide public health problem and is often treated by non-nephrologists. The objective of this study was to evaluate the knowledge of non-nephrologists, undergraduate medical students and health professionals regarding AKI.

**Methods:**

An unsupervised and closed-response electronic questionnaire was sent to sixth-year medical students and non-nephrologist health professionals working in the city of São Paulo, Brazil.

**Results:**

A total of 424 responses were returned from 650 invitations (40.1% males, 39.2% physicians, 34.0% senior medical students or resident physicians, 16.3% nurses and 10.6% pharmacists). The knowledge of medical students and health professionals about the prevalence and lethality of AKI in hospitals ranged from 40.8% to 59.2%. The most recognized susceptibilities and risk factors for AKI were sepsis/septic shock (95.0%) and diabetes mellitus (91.3%); the less-recognized susceptibilities and risk factors were exposure to gadolinium-based contrast (23.3%) and chronic liver disease (55.7%). The study participants’ rate of knowledge regarding the diagnosis of AKI was 50.9–73.6%, and their rate of knowledge regarding the criteria of increased serum creatinine and reduced urine volume was 52.6%. The most-recognized nephrotoxic medications were vancomycin (82.3%) and diclofenac (80.4%), and the least-recognized were acyclovir (34.0%) and cotrimoxazole (30.4%). The indications for emergency renal replacement therapy that were recognized by the study respondents were metabolic acidosis (82.3%), uremic syndrome (81.6%), hyperkalemia unresponsive to clinical treatment (78.1%) and acute pulmonary edema (71.6%).

**Conclusion:**

The study showed knowledge gaps that can impact patient care and can be used to guide professional training programs.

## Introduction

Acute kidney injury (AKI), characterized by the sudden worsening of renal function, is a global public health problem [1]. One in five hospitalized patients has AKI, and in intensive care, this prevalence reaches 50% [2]. The overall mortality of AKI is 20%, and when patients require renal replacement therapy (RRT), the risk of death increases to 60% [3]. AKI increases the mean hospitalization time by five days and increases hospital costs by more than 95% [3]. In addition, compared with patients without AKI, individuals with this syndrome have a ninefold increase in the risk of chronic kidney disease (CKD) and a threefold increase in the risk of end-stage CKD [4].

A bulletin of the World Health Organization that considered the United Nations Sustainable Development Goals (SDG) defined kidney diseases as one of the most neglected noncommunicable conditions in the world [5]. Professionals from many medical specialties encounter patients at risk for AKI. Studies have shown AKI incidences of 50% to 68% in sepsis patients, 13% to 60% in patients hospitalized for heart failure, 36% to 53% in pneumonia patients, 10% to 50% in patients after cardiac surgery and 48% in patients with previous CKD [6].

Audits performed in hospitals in the United Kingdom showed that most patients who died due to AKI did not receive adequate renal care [7]. In fact, simple and important measures such as fluid replacement, the discontinuation of nephrotoxic drugs and the correction of electrolyte disorders were performed in less than 50% of hospitalized patients with AKI [8,9].

Previous studies have shown that there are deficiencies in health professionals’ knowledge about the risk factors, prevention and basic treatment of AKI [10–13]. Considering that most cases of AKI are treated by non-nephrologists [9–14], assessing the knowledge of non-nephrologists and multidisciplinary health teams regarding AKI is essential for the development of training and care protocols.

Brazil is an upper-middle-income country located in Latin America with an estimated frequency of in-hospitals AKI of up to 20% [1,2]. AKI in Brazil has mixed characteristics: aging population, multiple comorbidities and highly complex procedures, and also lack of basic sanitation, poverty, infectious diseases and poisons from venomous animals [1,5]. As studies with this scope are scarce in our environment, the objective of this study was to evaluate the knowledge of non-nephrologists and undergraduate medical students regarding the risk factors for and the identification and management of AKI.

## Methods

### Design, population and elaboration of the questionnaire

The study was cross-sectional and was based on closed-ended questionnaire. The questionnaire was sent *via* a mobile phone application to 240 sixth-year medical students, 40 clinical medicine residents, 30 surgical residents, 210 non-nephrologists with various specialties, 80 nurses and 50 pharmacists. Among the medical students, only those who were in the sixth year of the course were included in order to assess the knowledge about AKI that was accumulated during medical school. The medical course in Brazil lasts for six years. Medical students and health professionals affiliated with public and private health institutions located in the city of São Paulo were included in the study. The participants and their institutions were selected for convenience, that is, they were part of the authors’ contact network. This type of sampling was chosen in order to obtain a higher response rate. The study was approved by the Research Ethics Committee of Universidade Nove de Julho under the certificate of presentation of ethical assessment (CAAE) number 29351120.1.0000.5511. Due to the characteristics of the study, it was exempted from applying an informed consent form.

The AKI questionnaire was performed in Portuguese and consisted of four parts: epidemiology, identification and risk factors, prevention and treatment and demographic data (Box 1). We chose not to include questions about the new AKI biomarkers in order to keep the questionnaire concise and focused in the general topics more relevant to the routine of a non-nephrologist health professional. The questions were prepared based on current guidelines [15]. Simple and familiar words were used, no acronyms or abbreviations were used, and no ambiguous questions were asked that included emotional content or conveyed approval or disapproval. The quality of the questionnaire was tested with an individual representant from each level and field training to verify assess the clarity of the statements, the importance of the questions and the level of fatigue after completing the questionnaire (Supplementary online material).

Box 1. Structure of the acute kidney injury questionnaire.

**Table:** 

Section	Question topic
1. Epidemiology	Prevalence
	Lethality
	Impact in the short and long term
2. Identification and risk factors	Susceptibilities
	Risk factors
	Diagnosis
3. Prevention and treatment	Preventive measures
	Nephrotoxic medications
	Indications for renal replacement therapy
4. Demographic data	Current role (medical student, doctor, nurse or pharmacist)
	Age, gender, year of graduation
	Specialty, postgraduate level
	Nature of the institution in which the respondent operates (public, private or both)
	Main type of activity (assistance or management)

### Statistical analysis

Assuming a correct response rate of 60%, a margin of error of 12% (20% of the estimate) and a confidence interval of 95%, we aimed to include 64 participants from each professional group [11–13]. Statistical analysis was performed using SPSS software, version 18.0 (SPSS Inc., Chicago, IL, USA). The categorical variables are described as frequencies. The quantitative variables are expressed as medians (interquartiles) because they exhibited a nonnormal distribution. A bicaudal p value <0.05 was considered significant. The comparison of frequencies was performed using the chi-square test with adjustment by the Bonferroni method. This analysis compares all possible combinations between groups. The subsets of participants whose knowledge levels did not differ significantly at the 0.05 level were identified by a letter.

## Results

Between 09/15/2020 and 05/29/2021, the study received valid responses from 424 participants with the following characteristics: 40.1% (170) males, age 31 (23–43) years, length of professional experience 8 (0–8) years, 39.2% (166) physicians, 22.9% (97) medical students, 16.3% (69) nurses, 11.1% (47) resident physicians and 10.6% (45) pharmacists. Answers from third-year medical students (3) and nephrologists (2) were excluded. Among health professional participants, the main areas of expertise were clinical medicine (10.1%), intensive care (10.1%), cardiology (6.1%) and general surgery (5.4%). The highest education level of the participants was specialization or residency, 54.2%; master’s degree, 8.7%; and doctorate, 6.6% (30.4% had no graduate degree). A total of 39.4%, 39.6% and 21.0% of the respondents worked in public institutions, private institutions and both. The participants’ main types of activity were direct patient care (80.4%) and management (19.6%) (Table 1).

**Table 1.** Characteristics of the study participants (*n* = 424).

A	
Variables	All (*n* = 424)
Age (years)	
20–24, % (*n*)	56 (13.2)
25–29, % (*n*)	95 (22.4)
30–34, % (*n*)	74 (17.5)
35–39, % (*n*)	37 (8.7)
40–44, % (*n*)	76 (17.9)
45–49, % (*n*)	52 (12.3)
50 or older, % (*n*)	34 (8.0)
Male gender, % (*n*)	170 (40.1)
Role	
Medical student, % (*n*)	97 (22.9)
Nurse, % (*n*)	69 (16.3)
Pharmacist, % (*n*)	46 (10.6)
Resident physician in clinical medicine, % (*n*)	24 (5.7)
Resident physician in surgery, % (*n*)	23 (5.4)
Attending physician or manager, % (*n*)	166 (39.2)
Main area of activity	
Student, % (*n*)	97 (22.9)
Cardiology, % (*n*)	26 (6.1)
General surgery, % (*n*)	23 (5.4)
Vascular surgery, % (*n*)	4 (0.9)
Internal medicine, % (*n*)	43 (10.1)
Dermatology, % (*n*)	6 (1.4)
Emergency, % (*n*)	4 (0.9)
Gynecology/obstetrics, % (*n*)	9 (2.1)
Infectious diseases, % (*n*)	4 (0.9)
Neurology, % (*n*)	4 (0.9)
Oncohematology, % (*n*)	11 (2.6)
Pulmonology, % (*n*)	2 (0.5)
Rheumatology, % (*n*)	2 (0.5)
Intensive care, % (*n*)	43 (10.1)
Urology, % (*n*)	3 (0.7)
Other, % (*n*)	116 (27.4)
Graduate study	
None, % (*n*)	129 (30.4)
Specialization or residency, % (*n*)	230 (54.2)
Master’s degree, % (*n*)	37 (8.7)
PhD, % (*n*)	28 (6.6)
Length of professional experience (years)	8 (0-8)
Nature of the institution in which the respondent operates	
Public, % (*n*)	167 (39.4)
Private, % (*n*)	168 (39.6)
Both, % (*n*)	89 (21.0)
Main type of activity	
Direct patient care, % (*n*)	341 (80.4)
Management, % (*n*)	83 (19.6)

B					
Variables	All (*n* = 424)	Medical students and residents (*n* = 144)	Clinical physicians (*n* = 99)	Surgeons (*n* = 67)	Nurses and pharmacists (*n* = 114)
Age (years)					
20–24, % (*n*)	56 (13.2)	49 (34.0)	2 (2.0)	1 (1.5)	4 (3.5)
25–29, % (*n*)	95 (22.4)	68 (47.2)	8 (8.1)	3 (4.5)	16 (14.0)
30–34, % (*n*)	74 (17.5)	19 (13.2)	19 (19.2)	15 (22.4)	21 (18.4)
35–39, % (*n*)	37 (8.7)	1 (0.7)	14 (14.1)	5 (7.5)	17 (14.9)
40–44, % (*n*)	76 (17.9)	3 (2.1)	20 (20.2)	25 (37.3)	28 (24.6)
45–49, % (*n*)	52 (12.3)	2 (1.4)	21 (21.2)	12 (17.9)	17 (14.9)
50 or older, % (*n*)	34 (8.0)	2 (1.4)	15 (15.2)	6 (9.0)	11 (9.6)
Male gender, % (*n*)	170 (40.1)	57 (39.6)	44 (44.4)	39 (58.2)	30 (26.3)
Role					
Medical student, % (*n*)	97 (22.9)	–	–	–	–
Nurse, % (*n*)	69 (16.3)	–	–	–	–
Pharmacist, % (*n*)	46 (10.6)	–	–	–	–
Resident physician in clinical medicine, % (*n*)	24 (5.7)	–	–	–	–
Resident physician in surgery, % (*n*)	23 (5.4)	–	–	–	–
Attending physician or manager, % (*n*)	166 (39.2)	–	–	–	–
Main area of activity					
Student, % (*n*)	97 (22.9)	–	–	–	–
Cardiology, % (*n*)	26 (6.1)	–	–	–	–
General surgery, % (*n*)	23 (5.4)	–	–	–	–
Vascular surgery, % (*n*)	4 (0.9)	–	–	–	–
Internal medicine, % (*n*)	43 (10.1)	–	–	–	–
Dermatology, % (*n*)	6 (1.4)	–	–	–	–
Emergency, % (*n*)	4 (0.9)	–	–	–	–
Gynecology/obstetrics, % (*n*)	9 (2.1)	–	–	–	–
Infectious diseases, % (*n*)	4 (0.9)	–	–	–	–
Neurology, % (*n*)	4 (0.9)	–	–	–	–
Oncohematology, % (*n*)	11 (2.6)	–	–	–	–
Pulmonology, % (*n*)	2 (0.5)	–	–	–	–
Rheumatology, % (*n*)	2 (0.5)	–	–	–	–
Intensive care, % (*n*)	43 (10.1)	–	–	–	–
Urology, % (*n*)	3 (0.7)	–	–	–	–
Other, % (*n*)	116 (27.4)	–	–	–	–
Graduate study					
None, % (*n*)	129 (30.4)	104 (72.2)	11 (11.1)	2 (3.0)	12 (10.5)
Specialization or residency, % (*n*)	230 (54.2)	38 (26.4)	59 (59.6)	45 (67.2)	88 (77.2)
Master’s degree, % (*n*)	37 (8.7)	1 (0.7)	14 (14.1)	13 (19.4)	9 (7.9)
PhD, % (*n*)	28 (6.6)	1 (0.7)	15 (15.2)	7 (10.4)	5 (4.4)
Length of professional experience (years)	8 (0-20)	0 (0-3)	19 (8-24)	20 (8-24)	12 (7-20)
Nature of the institution in which the respondent operates					
Public, % (*n*)	167 (39.4)	70 (48.6)	32 (32.3)	13 (19.4)	52 (45.6)
Private, % (*n*)	168 (39.6)	58 (40.3)	32 (32.3)	31 (46.3)	47 (41.2)
Both, % (*n*)	89 (21.0)	16 (11.1)	35 (35.4)	23 (34.3)	15 (13.2)
Main type of activity					
Direct patient care, % (*n*)	341 (80.4)	139 (96.5)	83 (83.8)	45 (67.2)	74 (64.9)
Management, % (*n*)	83 (19.6)	5 (3.5)	16 (16.2)	22 (32.8)	40 (35.1)

Data are presented as the number (percentage) or median (interquartile).

The participants’ rate of knowledge regarding the epidemiology of AKI in hospitals (prevalence and lethality) ranged from 40.8% to 59.2% and did not differ significantly according to their role. The impact of AKI on increasing the risk of nosocomial infection, the length of hospital stay, readmission, CKD risk and the long-term risk of cardiovascular disease was recognized by 54.2%, 81.8%, 59.0%, 76.4% and 1.7% of the participants, respectively. The knowledge that AKI has an effect on hospitalization time was lower among nurses and pharmacists than among medical students/residents and clinical physicians (70.2% vs. 86.1% vs. 88.9%, respectively) and similar to that of surgeons (70.2% vs. 82.1%). The impact of AKI on the risk of hospital readmission was less recognized by nurses/pharmacists than by medical students/residents (43.9% vs. 70.8%) and equally recognized by clinical physicians and surgeons (60.6% vs. 56.7%) (Table 2).

**Table 2.** Identification of the prevalence and lethality rates and impact of acute kidney injury by medical students/residents and health professionals (*n* = 424).

	Medical students and residents (*n* = 144)	Clinical physicians (*n* = 99)	Surgeons (*n* = 67)	Nurses and pharmacists (*n* = 114)	All (*n* = 424)
Prevalence rate					
AKI in hospitals	61_a_ (42.4)	43_a_ (43.4)	22_a_ (32.8)	47_a_ (41.2)	173 (40.8)
AKI in intensive care units	88_a_ (61.1)	54_a_ (54.5)	33_a_ (49.3)	54_a_ (47.4)	229 (54.0)
Mortality rate					
AKI in general	69_a_ (47.9)	34_a_ (34.3)	26_a_ (38.8)	46_a_ (40.4)	175 (41.3)
AKI requiring dialysis	86_a_ (59.7)	65_a_ (65.7)	45_a_ (67.2)	55_a_ (48.2)	251 (59.2)
Impact of AKI					
Increased risk of infection	83_a_ (57.6)	53_a_ (53.5)	40_a_ (59.7)	54_a_ (47.4)	230 (54.2)
Increased length of hospital stay	124_a_ (86.1)	88_a_ (88.9)	55_a,b_ (82.1)	80_b_ (70.2)	347 (81.8)
Increased risk of hospital readmission	102_a_ (70.8)	60_a,b_ (60.6)	38_a,b_ (56.7)	50_b_ (43.9)	250 (59.0)
Increased risk of cardiovascular disease	6_a_ (4.2)	1_a_ (1.0)	0_a_ (0.0)	0_a_ (0.0)	7 (1.7)
Increased risk of chronic kidney disease	114_a_ (79.2)	69_a_ (69.7)	54_a_ (80.6)	87_a_ (76.3)	324 (76.4)

Data are presented as the number of correct answers (percentage). Each subscript letter indicates a subset of participants whose knowledge levels do not differ significantly at the 0.05 level. AKI: acute kidney injury.

The most frequently recognized susceptibilities and risk factors for AKI were sepsis/septic shock (95.0%), diabetes mellitus (91.3%), dehydration (90.8%) and the use of iodine-based radiological contrasts (88, 4%), while the less frequently identified were bradycardia (23.3%), hypervolemia (23.3%), the use of gadolinium-based contrasts (23.3%) and chronic liver disease (55.7%). The participants that recognized proteinuria as a susceptibility to AKI were 73.8% (67.7–80.6%). This percentage did not differ significantly among the groups of survey respondents. The identification of hypovolemia, hypotension, sepsis/septic shock and moderate and major surgery as risk factors/susceptibilities was lower among nurses/pharmacists (74.6%, 39.5%, 83.3% and 48.2%, respectively) than among the other participants (91.9–93.8%, 62.5–74.7%, 98.6–100% and 68.7–83.8%, respectively). Nurses/pharmacists and surgeons also recognized dehydration as a risk factor for AKI (88.1% and 78.9%, respectively), but did so in lower proportions than clinical physicians (99.0%) (Table 3).

**Table 3.** Identification of susceptibilities, risk factors, preventive measures and diagnostic criteria for acute kidney injury by medical students/residents and health professionals (*n* = 424).

	Medical students and residents (*n* = 144)	Clinical physicians (*n* = 99)	Surgeons (*n* = 67)	Nurses and pharmacists (*n* = 114)	All (*n* = 424)
Susceptibilities					
Advanced age	124_a_ (86.1)	80_a_ (80.8)	50_a,b_ (74.6)	67_b_ (58.8)	321 (75.7)
Diabetes mellitus	135_a_ (93.8)	92_a_ (92.9)	63_a_ (94.0)	97_a_ (85.1)	387 (91.3)
Heart failure	95_a,b_ (66.0)	79_b_ (79.8)	55_b_ (82.1)	71_a_ (62.3)	300 (70.8)
Chronic liver disease	82_a,b_ (56.9)	63_b_ (63.6)	43_b_ (64.2)	48_a_ (42.1)	236 (55.7)
eGF*R* < 60 ml/min	104_a_ (72.2)	78_a_ (78.8)	46_a_ (68.7)	71_a_ (62.3)	299 (70.5)
Proteinuria	108_a_ (75.0)	67_a_ (67.7)	54_a_ (80.6)	84_a_ (73.7)	313 (73.8)
Risk factors					
Arterial hypotension	90_a_ (62.5)	74_a_ (74.7)	50_a_ (74.6)	45_b_ (39.5)	259 (61.1)
Bradycardia	33_a_ (22.9)	26_a_ (26.3)	18_a_ (26.9)	22_a_ (19.3)	99 (23.3)
Dehydration	138_a,b_ (95.8)	98_b_ (99.0)	59_a,c_ (88.1)	90_c_ (78.9)	385 (90.8)
Hypovolemia	135_a_ (93.8)	91_a_ (91.9)	62_a_ (92.5)	85_b_ (74.6)	373 (88.0)
Hypervolemia	40_a_ (27.8)	18_a_ (18.2)	14_a_ (20.9)	27_a_ (23.7)	99 (23.3)
Moderate or major surgeries	109_a_ (75.7)	83_a_ (83.8)	46_a_ (68.7)	55_b_ (48.2)	293 (69.1)
Iodine-based radiological contrast	133_a_ (92.4)	92_a_ (92.9)	61_a,b_ (91.0)	89_b_ (78.1)	375 (88.4)
Gadolinium-based radiological contrast	31_a,b_ (21.5)	14_b_ (14.1)	20_a,b_ (29.9)	34_a_ (29.8)	99 (23.3)
Infections	122_a_ (84.7)	82_a_ (82.8)	55_a_ (82.1)	83_a_ (72.8)	342 (80.7)
Sepsis or septic shock	142_a_ (98.6)	99_a_ (100.0)	67_a_ (100.0)	95_b_ (83.3)	403 (95.0)
Diagnostic criteria					
Creatinine increas*e* ≥ 0.3 mg/dl compared to baseline in up to 48 h	126_a_ (87.5)	58_b_ (58.6)	40_b,c_ (59.7)	88_a,c_ (77.2)	312 (73.6)
Creatinine increas*e* ≥ 50% compared to baseline in up to 7 days	81_a_ (56.3)	52_a_ (52.5)	34_a_ (50.7)	49_a_ (43.0)	216 (50.9)
Diuresi*s* < 0.5 ml/kg/h for more than 6 h	91_a_ (63.2)	55_a,b_ (55.6)	31_a,b_ (46.3)	46_b_ (40.4)	223 (52.6)
Preventive measures					
Correction of hypotension	107_a_ (74.3)	82_a_ (82.8)	56_a_ (83.6)	63_b_ (55.3)	308 (72.6)
Hydration	139_a_ (96.5)	97_a_ (98.0)	63_a,b_ (94.0)	99_b_ (86.8)	398 (93.9)
Use of vasoactive drugs in cases of shock	86_a_ (59.7)	56_a_ (56.6)	29_a,b_ (43.3)	33_b_ (28.9)	204 (48.1)
Recognition that dopamine has no nephroprotective effect on AKI	128_a_ (88.9)	81_a_ (81.8)	56_a_ (83.6)	100_a_ (87.7)	365 (86.1)
Recognition that diuretics should not be routinely used for the treatment of oliguria	104_a_ (72.2)	86_b_ (86.9)	51_a,b_ (76.1)	73_a_ (64.0)	314 (74.1)

Data are presented as the number of correct answers (percentage). Each subscript letter indicates a subset of participants whose knowledge levels do not differ significantly at the 0.05 level. eGFR, estimated glomerular filtration rate; AKI, acute kidney injury.

A total of 73.6%, 50.9% and 52.6% of the study participants recognized a serum creatinine increase ≥0.3 mg/dl within 48 h, a creatinine increase ≥50% in up to 7 days and a urine volume ≤0.5 mL/kg/hour for more than 6 h, respectively, as criteria for AKI diagnosis [15]. Clinical physicians and surgeons were less familiar with the AKI criterion for creatinine variation at 48 h than medical students/residents were (58.6–59.7% vs. 87.5%). A total of 72.6%, 93.9% and 48.1% of the respondents recognized that the correction of arterial hypotension, hydration and the use of vasoactive drugs in cases of shock, respectively, are preventive measures for AKI. Among the study participants, 86.1% and 74.1% recognized that dopamine and the routine use of diuretics, respectively, have no protective effect against AKI. There was no significant difference in the knowledge of medical students/residents, clinical physicians and surgeons regarding the use of preventive measures for AKI (Table 3).

The nephrotoxic medications recognized by the survey respondents were (in descending order) vancomycin (82.3%), diclofenac (80.4%), amphotericin (71.5%), amikacin (71.2%), ibuprofen (71, 2%), gentamicin (64.6%), polymyxin (63.7%), cyclosporine (50.5%), cisplatin (41.0%), etoricoxib (41.0%), tenofovir (40.1%), acyclovir (34.0%) and cotrimoxazole (30.4%) (Table 4).

**Table 4.** Identification of nephrotoxic drugs by medical students/residents and health professionals (*n* = 424).

Medication	Medical students and residents (*n* = 144)	Clinical physicians (*n* = 99)	Physicians and surgeons (*n* = 67)	Nurses (*n* = 69)	Pharmacists (*n* = 45)	All (*n* = 424)
Acyclovir	46_a_ (31.9)	34_a_ (34.3)	23_a_ (34.3)	21_a_ (30.4)	20_a_ (44.4)	144 (34.0)
Amikacin	101_a_ (70.1)	85_b_ (85.9)	49_a,b_ (73.1)	34_c_ (49.3)	33_a,b. c_ (73.3)	302 (71.2)
Amphotericin	105_a,b_ (72.9)	81_b_ (81.8)	44_a,b_ (65.7)	43_a_ (62.3)	30_a,b_ (66.7)	303 (71.5)
Cyclosporine	77_a_ (53.5)	54_a_ (54.5)	36_a_ (53.7)	27_a_ (39.1)	20_a_ (44.4)	214 (50.5)
Cisplatin	61_a_ (42.4)	43_a_ (43.4)	29_a_ (43.3)	20_a_ (29.0)	21_a_ (46.7)	174 (41.0)
Cotrimoxazole	52_a_ (36.1)	32_a_ (32.3)	13_a_ (19.4)	15_a_ (21.7)	17_a_ (37.8)	129 (30.4)
Diclofenac	125_a_ (86.8)	97_b_ (98.0)	52_a_ (77.6)	51_a_ (73.9)	31_a_ (68.9)	356 (84.0)
Etoricoxib	54_a,b.c.d.e_ (37.5)	54_d. e_ (54.5)	31_c. e_ (46.3)	14_b_ (20.3)	21_a,c.d.e_ (46.7)	174 (41.0)
Gentamicin	91_a_ (63.2)	80_b_ (80.8)	51_a,b_ (76.1)	24_c_ (34.8)	28_a,b_ (62.2)	274 (64.6)
Ibuprofen	113_a_ (78.5)	84_a_ (84.8)	54_a_ (80.6)	31_b_ (44.9)	20_b_ (44.4)	302 (71.2)
Polymyxin	84_a_ (58.3)	76_b_ (76.8)	40_a,b_ (59.7)	47_a,b_ (68.1)	23_a_ (51.1)	270 (63.7)
Tenofovir	77_a_ (53.5)	43_a,b_ (43.4)	22_a,b_ (32.8)	16_b_ (23.2)	12_b_ (26.7)	170 (40.1)
Vancomycin	106_a_ (73.6)	94_b_ (94.9)	60_a,b_ (89.6)	53_a_ (76.8)	36_a,b_ (80.0)	349 (82.3)

Data are presented as the number of correct answers (percentage). Each subscript letter indicates a subset of participants whose knowledge levels do not differ significantly at the 0.05 level.

Pharmacists identified amikacin as nephrotoxic in a proportion similar to that of students/residents, clinical physicians and surgeons (73.7% vs. 70.1–85.9%). The knowledge that diclofenac is nephrotoxic was greater among clinical physicians than among students/residents, surgeons and pharmacists (98.0% vs. 68.9–86.8%). The proportion of clinical physicians that identified gentamicin as nephrotoxic was higher than the proportion students/residents (80.8%vs. 63.2%) but similar to the proportions of surgeons (76.1%) and pharmacists (62.2%). The knowledge that ibuprofen is nephrotoxic was lower among pharmacists than among students/residents, clinical physicians and surgeons (44.4% vs. 78.5–84.8%). The proportion of clinical physicians that identified polymyxin as nephrotoxic was higher than that of students/residents and pharmacists (76.8% vs. 51.1–58.3%) and similar to that of surgeons (59.7%). The knowledge that tenofovir is nephrotoxic was higher among students/residents than pharmacists (53.5% vs. 26.7%) and similar to that of clinical physicians (43.4%) and surgeons (32.8%). Clinical physicians identified vancomycin as nephrotoxic in a higher proportion than students/residents (94.9% vs. 73.6%) and similar to that of surgeons (89.6%) and pharmacists (80.0%). There was no difference in knowledge regarding the nephrotoxicity of acyclovir, cisplatin, cyclosporine and cotrimoxazole among the different groups of study participants (Table 4).

The indications for emergency RRT that were recognized by the study respondents were (in descending order) metabolic acidosis (82.3%), uremic syndrome (81.6%), hyperkalemia resistant to clinical treatment (78.1%), acute edema of the lungs (71.6%), lithium intoxication (57.7%) and malignant hypercalcemia (44.8%). Clinical physicians identified hyperkalemia as an indication for emergency RRT more frequently than medical surgeons (85.9% vs. 68.7%) and in an equal proportion to students/residents. The knowledge that lithium poisoning may be an indication for emergency RRT was higher among surgeons than among students/residents (71.6% vs. 48.6%) and was similar to that of clinical physicians (61.6%). There was no difference in the knowledge that metabolic acidosis, uremic syndrome and malignant hypercalcemia are indications for emergency RRT among the different study participant groups (Table 5).

**Table 5.** Identification of indications for emergency RRT by medical students/residents and medical professionals (*n* = 310).

	Medical students and residents (*n* = 144)	Clinical physicians (*n* = 99)	Physicians and surgeons (*n* = 67)	All (*n* = 310)
Acute pulmonary edema	96_a_ (66.7)	79_a_ (79.8)	47_a_ (70.1)	222 (71.6)
Hyperkalemia	111_a,b_ (77.1)	85_b_ (85.9)	46_a,c_ (68.7)	242 (78.1)
Metabolic acidosis	123_a_ (85.4)	79_a_ (79.8)	53_a_ (79.1)	255 (82.3)
Uremic syndrome	113_a_ (78.5)	83_a_ (83.8)	57_a_ (85.1)	253 (81.6)
Malignant hypercalcemia	66_a_ (45.8)	46_a_ (46.5)	27_a_ (40.3)	139 (44.8)
Lithium poisoning	70_a,b_ (48.6)	61_b,c_ (61.6)	48_c_ (71.6)	179 (57.7)

Data are presented as the number of correct answers (percentage). Each subscript letter indicates a subset of participants whose knowledge levels do not differ significantly at the 0.05 level. RRT, renal replacement therapy.

## Discussion

The present study showed important gaps in the knowledge about AKI among medical students, residents and health professionals in the municipality of São Paulo. A lack of knowledge was observed in all topics analyzed, that is, epidemiology, risk factors and susceptibilities, nephrotoxic drugs and basic treatment of AKI.

The medical students and resident physicians who participated in this study knew more about the diagnostic criteria for AKI than the physicians who had completed their training, which could be explained by the fact that the most current definitions of AKI are relatively recent [15]. The lack of knowledge regarding the definition of AKI according to the current criteria of increased creatinine and reduced diuresis volume may impact the early diagnosis of this condition. After the standardizing of the definition of AKI, studies have shown a significant increase in AKI diagnostic records in several countries [16]. Nevertheless, the coded incidence of AKI remains lower than the real incidence [17,18].

The actual prevalence and severity of this condition were unknown by approximately half of the respondents, regardless of their area of practice. Along this line, a previous study showed Brazilian nurses’ knowledge regarding the incidence of AKI was 54.6% [13]. The health professionals’ knowledge regarding the lethality of RRT-dependent AKI that was observed in this study was greater than that reported in a previous study (59.2% versus 13.0%) [13]. The study period could explain this difference. The present study was conducted during the pandemic of SARS-CoV-2, a pathogen with a high incidence of kidney involvement, which increased the prevalence and lethality of patients who were on RRT due to AKI [19]. Indeed, the impact of AKI on the mortality of patients with and without COVID-19 has been recently addressed in Brazilian multicenter studies [20–22].

Most of the assessed risk factors and susceptibilities for AKI were recognized by more than 70% of participants, with the exception of the low knowledge regarding the risk of AKI associated with chronic liver disease and the use of gadolinium-based contrasts. A previous study showed that the risk factors for AKI that were less recognized among Nigerian physicians were chronic liver disease and advanced age [11]. The greater knowledge that elderly adults are at a higher risk of AKI among the participants in our study can be explained by geographical differences. Compared to urbanized regions, in many regions of the African continent, AKI is usually acquired in the community and more often affects young and previously healthy individuals [23].

In this study, it was noted that nurses and pharmacists had lower levels of knowledge than medical professionals regarding some risk factors and susceptibilities, suggesting the need to provide AKI training opportunities for all health professionals. One explanation for this result would be that these professionals held more management positions than the other groups of participants (and consequently would be less involved with direct patient care). A lack of identification of patients at risk for AKI may reduce the likelihood that simple preventive measures, such as urine output monitoring and body weight control, are performed [7].

More than 70% of the participants in this study recognized proteinuria as susceptibility to AKI. Previous studies on the knowledge of health professionals about AKI did not assess this variable [7–13]. The result found in our study could be considered satisfactory when compared to the low rate of proteinuria testing in people with arterial hypertension and diabetes mellitus [24,25]. It is well known that proteinuria is a risk factor of worse prognosis for patients with CKD and a risk factor for the development and worse outcomes of AKI [15,26].

In general, the recognition that drugs that are commonly used in hospitals are nephrotoxic was unsatisfactory, a finding that corroborates previous studies [11–13]. Clinical physicians’ knowledge regarding some of these drugs (diclofenac, gentamicin and polymyxin) was greater than that of medical students and residents, which could indicate that the topic is not properly addressed at the undergraduate level. However, we did not find studies or evaluate the curriculum of universities to confirm this hypothesis. In addition, for some medications (diclofenac, ibuprofen, polymyxin and tenofovir), pharmacists had lower levels of knowledge than medical professionals did, which could suggest a lack of pharmaceutical intervention in the institutions where the study participants worked. As mentioned above, another hypothesis for this result would be the lower percentage of allocation of these professionals in direct health care. In any case, it is important to note that studies have shown that the active participation of pharmacists in reviewing medical prescriptions in the hospital environment is associated with fewer adverse drug events and lower costs [27–29].

Common indications for emergency RRT, such as acute lung edema and hyperkalemia that is unresponsive to clinical treatment, were not identified by up to 33% of the professionals with medical training. This fact should be carefully noted because the delay in communicating with nephrologists, especially in severe cases of AKI, was associated with higher mortality in several studies [30].

Some limitations of this study should be recognized, such as the relatively small sample of pharmacists and resident physicians and the inability to generalize the conclusions to other municipalities in the state of São Paulo or Brazil. In addition, sending the questionnaire electronically to people in the authors’ network may have constituted a selection bias. For ethical reasons, the questionnaire did not include any items that could identify the institutions with which the students and health professionals were affiliated, which made it impossible to create individual profiles of health services or schools. Nevertheless, this study was one of the largest and most complete surveys ever conducted in Brazil to assess knowledge about AKI.

## Conclusion

The study showed important gaps in the knowledge of AKI among medical students, residents and health professionals in the municipality of São Paulo. These deficiencies can negatively impact patient care, and awareness of them can guide professional training programs in efforts to reduce the incidence, severity and unfavorable outcomes of AKI.

## Supplementary Material

Supplemental Material
