# Supplementary material for: Medical students’ and health professionals’ knowledge regarding acute kidney injury: a cross-sectional study in the city of São Paulo, Brazil
Source: Ren Fail. 2022 Nov 3;44(1):1660–8. doi: 10.1080/0886022X.2022.2131575 (PMC9639479; doi:10.1080/0886022X.2022.2131575)
Supplement: Supplemental Material [file IRNF_A_2131575_SM5887.pdf]

Medical students and health professionals' knowledge regarding acute kidney injury

Supplementary material

Acute kidney injury questionnaire

The expected correct answers are marked by filled squares.

Section 1. Epidemiology

1. In adult population, how prevalent is acute kidney injury in hospitals?
   - ☐ 1%
   - ☐ 5%
   - ■ 20%
   - ☐ 30%
2. In general, what is the prevalence of acute kidney injury in intensive care units?
   - ☐ 5%
   - ☐ 10%
   - ■ 50%
   - ☐ 80%
3. What is the estimated mortality of patients with acute kidney injury in hospitals?
   - ☐ 0,5 to 1%
   - ■ 5 to 10%
   - ☐ 20 to 30%
   - ☐ 80 to 100%
4. What is the estimated mortality of patients with acute kidney injury requiring dialysis?
   - ☐ 1 to 2%
   - ☐ 5 to 10%
   - ■ 20 to 60%
   - ☐ 80 to 100%
5. What is the impact of the acute kidney injury? Tick the option (s) that you think is (are) correct:
   - ■ Increase the risk of nosocomial infection
   - ■ 2x increase in the length of stay and costs
   - ■ Increase the risk of hospital readmission by 20%

- ■ Increase the risk of long-term chronic kidney disease
- ■ Increase the risk of a long-term cardiovascular event

Section 2. Identification and risk factors

1. The following are risk factors for acute kidney injury. Tick the option (s) that you think is (are) correct:
   - ■ Arterial hypotension
   - ■ Bradycardia
   - ■ Dehydration
   - ■ Hypervolemia
   - ■ Hypovolemia
   - ■ Medium and major surgeries
   - ■ Iodine-based radiological contrast
   - ■ Gadolinium-based radiological contrast
   - ■ Infections
   - ■ Sepsis and septic shock
2. The following are susceptibilities to acute kidney injury are. Tick the option (s) that you think is (are) correct:
   - ■ Advanced age
   - ■ Diabetes mellitus
   - ■ Heart failure
   - ■ Chronic liver disease
   - ■ Estimated glomerular filtration rate < 60ml/min/1.73m^2
   - ■ Proteinuria
3. How do you define acute kidney injury? Tick the option (s) that you think is (are) correct:
   - ■ Creatinine increase ≥0.3mg/dl from baseline in <48h
   - ■ Creatinine increase ≥50% from baseline in <7 days
   - □ ≥50% increase in urea from baseline in <48h
   - ■ Diuresis <0.5ml/kg/h for ≥6 hours

Section 4. Prevention and treatment

1. What are the preventive measures against acute kidney injury? Tick the option (s) that you think is (are) correct:

■ Correction of arterial hypotension
■ Hydration
■ Vasoactive drug use in shock
□ Low dose dopamine
□ Routine use of diuretics for patients with oliguria

2. These are nephrotoxic drugs. Tick the option (s) that you think is (are) correct:

■ Acyclovir
■ Amikacin
■ Amphotericin
■ Cyclosporine
■ Cisplatin
■ Cotrimoxazole
■ Diclofenac
■ Etoricoxib
■ Gentamicin
■ Ibuprofen
■ Polymyxin
■ Tenofovir
■ Vancomycin

3. The following are indications for urgent dialysis. Tick the option (s) that you think is (are) correct:

■ Acute pulmonary edema
■ Hyperkalemia
■ Metabolic acidosis
■ Uremic syndrome
■ Malignant hypercalcemia
■ Lithium poisoning

AKI questionnaire: elaboration and validation steps

1. Drafting
2. Adjustments done by university professors of nephrology
3. Face-to-face validation with a professional from each group
4. Questions of the validation step: “What is your opinion about the acute kidney injury questionnaire?”

- Clarity of questions: ( ) good ( ) regular ( ) bad
- Importance of the topic: ( ) very important ( ) regular ( ) not important
- Fatigue level to answer: ( ) high ( ) regular ( ) low

References

1. Kidney Disease: Improving Global Outcomes (KDIGO). Acute Kidney Injury Work Group. KDIGO Clinical Practice Guideline for Acute Kidney Injury. Kidney Int Suppl. 2012;2:1-138
2. Saran S, Rao NS, Azim A. Drug Dosing in Critically Ill Patients with Acute Kidney Injury and on Renal Replacement Therapy. Indian J Crit Care Med. 2020;24(Suppl 3):S129-S134.

Acute kidney injury questionnaire in original language

As respostas corretas estão marcadas por quadrados cheios.

Seção 1. Epidemiologia

1. Qual a prevalência estimada de injuria renal aguda em hospitais?
   - ☐ 1%
   - ☐ 5%
   - ■ 20%
   - ☐ 30%
2. Qual a prevalência estimada de injúria renal aguda em unidades de terapia intensiva?
   - ☐ 5%
   - ☐ 10%
   - ■ 50%
   - ☐ 80%
3. Qual a mortalidade estimada de pacientes com injúria renal aguda em hospitais?
   - ☐ 0,5 to 1%
   - ■ 5 to 10%
   - ☐ 20 to 30%
   - ☐ 80 to 100%

4. Qual a mortalidade estimada de pacientes com injúria renal aguda que necessitam diálise em hospitais?
   - □ 1 to 2%
   - □ 5 to 10%
   - ■ 20 to 60%
   - □ 80 to 100%
5. Qual o impacto da injúria renal aguda? Assinale todas as opções que julgar corretas:
   - ■ Aumento do risco de infecção hospitalar
   - ■ Aumento de 2x no tempo de internação e nos custos hospitalares
   - ■ Aumento do risco de readmissão em 20%
   - ■ Aumento do risco de doença renal crônica em longo prazo
   - ■ Aumento do risco de evento cardiovascular em longo prazo

Seção 2. Identificação e fatores de risco

1. Os seguintes são fatores de risco para injúria renal aguda. Assinale toda as opções que julgar corretas:
   - ■ Hipotensão arterial
   - ■ Bradicardia
   - ■ Desidratação
   - ■ Hipervolemia
   - ■ Hipovolemia
   - ■ Cirurgias de médio e grande porte
   - ■ Contrastes iodados
   - ■ Contrastes à base de gadolínio
   - ■ Infecções
   - ■ Sepse e choque séptico

2. Os seguintes são susceptibilidade à injúria renal aguda. Assinale todas as opções que julgar corretas:
   - ■ Idade avançada
   - ■ Diabetes mellitus
   - ■ Insuficiência cardíaca
   - ■ Hepatopatia crônica

- ■ Taxa de filtração glomerular estimada < $60ml/min/1,73m^2$
- ■ Proteinúria

3. Como você define injuria renal aguda? Assinale todas as opções que julgar corretas:
   - ■ Aumento de creatinina ≥0,3mg/dl em relação ao basal em até 48h
   - ■ Aumento de creatinina ≥50% em relação ao basal em até 7 dias
   - □ Aumento de ureia ≥50% em relação ao basal em até 48h
   - ■ Volume de diurese <0,5ml/kg/h por ≥6 horas

Seção 4. Prevenção e tratamento

1. Quais dos seguintes são medidas preventivas contra injúria renal aguda? Assinale todas as opções que julgar corretas:
   - ■ Correção de hipotensão arterial
   - ■ Hidratação
   - ■ Droga vasoativa em pacientes com choque
   - □ Dose baixa de dopamina
   - □ Uso de diuréticos de rotina em pacientes com oligúria
2. Os seguintes são drogas nefrotóxicas. Assinale todas as opções que julgar corretas:
   - ■ Aciclovir
   - ■ Amicacina
   - ■ Anfotericina
   - ■ Ciclosporina
   - ■ Cisplatina
   - ■ Cotrimoxazol
   - ■ Diclofenaco
   - ■ Etoricoxibe
   - ■ Gentamicina
   - ■ Ibuprofeno
   - ■ Polimixina
   - ■ Tenofovir
   - ■ Vancomicina

3. Os seguintes são indicações de diálise de urgência. Assinale todas as opções que julgar corretas:
   - ■ Edema agudo de pulmão
   - ■ Hipercalemia
   - ■ Acidose metabólica
   - ■ Síndrome urêmica
   - ■ Hipercalcemia maligna
   - ■ Intoxicação por lítio

Questionário de Injúria Renal Aguda: etapas de elaboração e validação

1. Rascunho inicial
2. Ajustes realizados por professores de nefrologia
3. Validação presencial com um indivíduo de cada nível de formação
4. Perguntas realizadas nas etapas de validação: “Qual sua opinião sobre o questionário de injúria renal aguda?”

- Clareza das questões: ( ) bom ( ) regular ( ) ruim
- Importância do tema: ( ) muito importante ( ) regular ( ) não é importante
- Nível de fadiga para responder: ( ) alto ( ) regular ( ) baixo
